# Comparison of Texture Features Used for Classification of Life Stages of Malaria Parasite

**DOI:** 10.1155/2016/7214156

**Published:** 2016-05-09

**Authors:** Vinayak K. Bairagi, Kshipra C. Charpe

**Affiliations:** ^1^E & TC Engineering Department, AISSMS Institute of Information Technology, Pune 411001, India; ^2^Electronics Engineering Department, AISSMS Institute of Information Technology, Pune 411001, India

## Abstract

Malaria is a vector borne disease widely occurring at equatorial region. Even after decades of campaigning of malaria control, still today it is high mortality causing disease due to improper and late diagnosis. To prevent number of people getting affected by malaria, the diagnosis should be in early stage and accurate. This paper presents an automatic method for diagnosis of malaria parasite in the blood images. Image processing techniques are used for diagnosis of malaria parasite and to detect their stages. The diagnosis of parasite stages is done using features like statistical features and textural features of malaria parasite in blood images. This paper gives a comparison of the textural based features individually used and used in group together. The comparison is made by considering the accuracy, sensitivity, and specificity of the features for the same images in database.

## 1. Introduction

An infected female* Anopheles* mosquito is the host, which spread the malaria parasite [1]. Malaria is a highly occurring, vector borne disease in equatorial region. World Health Organization (WHO) has given the analysis report for the year 2013 as 300–500 billion people were the victims of malaria [1, 2]. There is a need for saving lives by making early diagnosis and medication. Nowadays in the biomedical field, all the disease diagnosis methods are turning into computerized automatic way. In this paper, diagnosis of malaria parasite in the blood images is made computerized by using the proposed method which works with image processing.

There are many clinical methods which can be used for malaria diagnosis which are peripheral blood smear (PBS), quantitative buffy coat (QBC), rapid diagnosis test (RDT), Polymerase Chain Reaction (PCR), and Third Harmonic Generation (THG) [3, 4]. Among these entire tests, the PBS is widely used and has limitations of human resistance and time requirement. These limitations can be overcome by making the system automated. The blood images are processed and then the diagnosis is done. The major problem in processing the blood images is the segmentation of red blood cells (RBC) from the background; a proper separation can help to achieve high accuracy in detecting the presence of plasmodium and helps in classification of life cycle stages of malaria parasite.

The life cycle stages of malaria parasite are differentiated into three different stages, that is, trophozoites, schizocytes, and gametocytes [3]. Each one of them is characterized by unique features such as shape, size, and texture. To identify and classify these stages, the above features will play important role while working with image processing. Features are the key way to differentiate and diagnose the parasite stages.

The organization of this paper is as follows.  Section 2 gives the detailed methodology about the work done and all the different image processing techniques used stepwise, while Section 3 provides all the comparison results obtained in presented study and lastly Section 4 gives the discussion followed by conclusion part of the complete research work carried out.

## 2. Methodology

**Figure 1:**
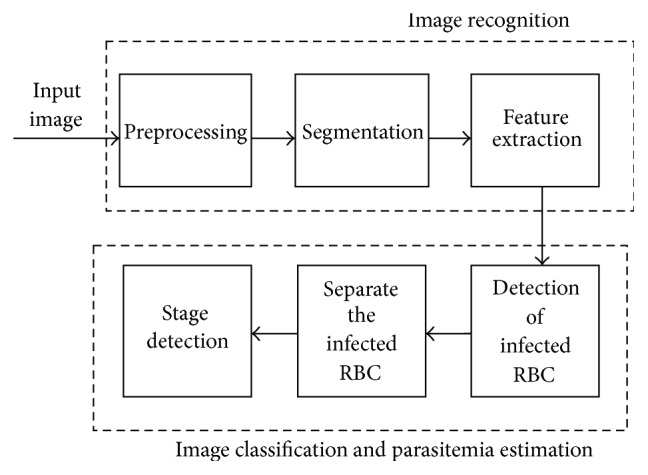
Block diagram of the proposed method.

The method of detection of malaria parasite is mainly subdivided into two parts first as the image recognition and second as the image classification and parasitemia estimation. The proposed method follows the stepwise work as shown in Figure 1, as block diagram.

The first block consists of the three basic steps of image processing, that is, preprocessing, segmentation, and feature extraction, while in the image classification and detection of infected red blood cells that is called parasitemia separation of infected RBC and the stage detection are carried out.

In this work, the malaria images of three different stages, schizont, trophozoite, and gametocyte stages, have been captured from the blood smears. First a database was created using images from the online database like Center for Disease Control (CDC) and Prevention and from Public Health Image Library [5] consisting of both types of samples parasitic and nonparasitic blood samples. Some images were added into the database which was obtained from Smt. Kashibai Navale General Hospital and Research Centre, Pune, consisting of both malaria positive blood samples and the normal blood samples images. The oil immersion images are taken from the digital camera with 100x zoom. Altogether, 100-image database was created among which 50 images are used for training stages and the remaining 50 images for testing stage were used.

### 2.1. Preprocessing

**Figure 2:**
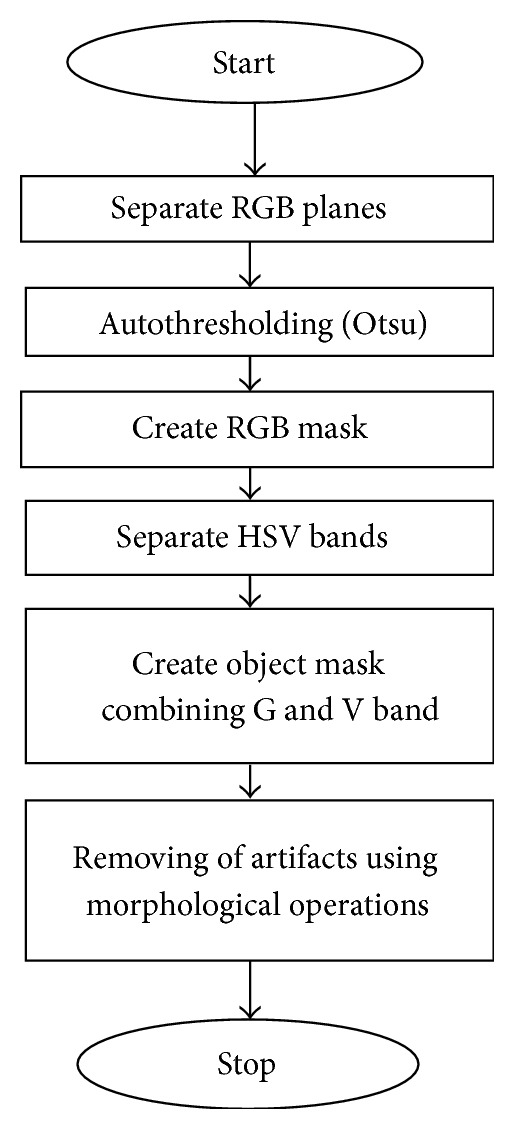
Flowchart for preprocessing.

Preprocessing is done to make the images easy to be processed further as it removes the noise and other unwanted artifact from the image under observation. The steps followed in preprocessing can be explained as in Figure 2 flowchart.

**Figure 3:**
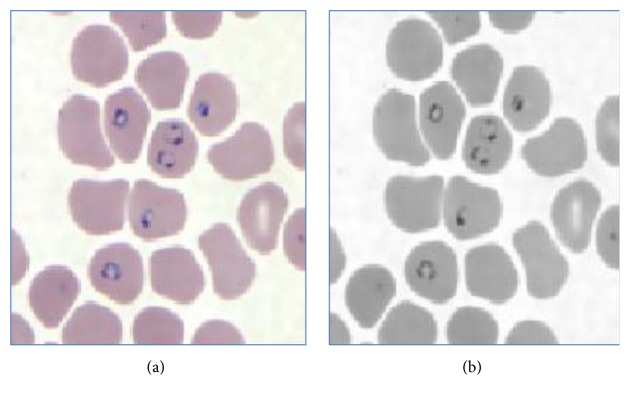
RGB image to gray scale image. (a) RGB image. (b) Gray image.

**Figure 4:**
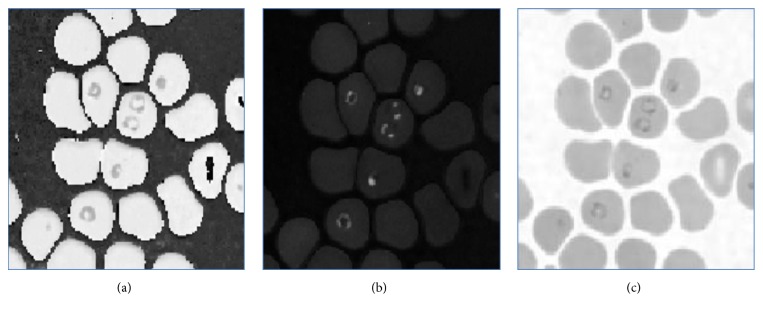
Separated HSV bands: (a) H band, (b) S band, and (c) V band.

**Figure 5:**
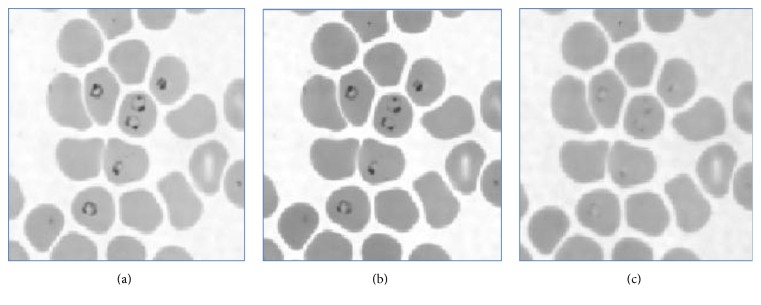
Separated RGB bands: (a) R band, (b) G band, and (c) B band.

**Figure 6:**
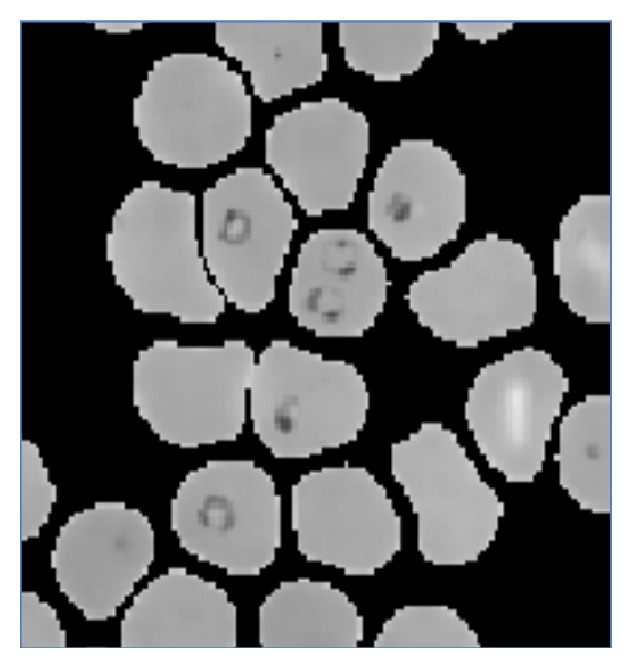
Gray scale converted image of object mask.

Initially the RGB image is converted into the gray image as shown in Figure 3. The basic Red, Green, and Blue (RGB) planes of the input image are separated so as to create object mask for segmentation. The RGB plane separation is done as shown in Figure 5. As there are intensity variations all over the image, to take histogram and work on intensity factor, thresholding is the key requirement. Therefore, autothresholding is done using Otsu's method [6]. An object mask is the image which is used as the standard sample to segment the region of interest in the input image and hence it should contain the parasitic part in the image. Therefore, considering all these aspects, the Hue, Saturation, and Value (HSV) bands are created, as shown in Figure 4. Combined GV band is obtained and named as object mask. This object mask is used for further process because it gives good quality image containing all the RBC and parasites clearly as shown in Figure 6. Finally, the morphological operations like closing and opening are performed on the object mask for removing the unwanted artifacts [7].

### 2.2. Segmentation

Segmentation is the process carried out to obtain the area of interest from the complete image [8]. As the malaria parasite affects the red blood cells [3, 4], the region of interest is the red blood cells in the microscopic images of blood sample. In this work, the segmentation is done to separate each red blood cell. These segmented cells are further processed and then the infected red blood cells are identified. The segmentation technique used in this work is watershed transform with the distance transform. The main reason of using watershed transform is to detect the overlapped red blood cells. The principal concept of watershed is to change the inputimage into another image whose catchment basins are the objects or regions which are required to be segmented or identified.

**Figure 7:**
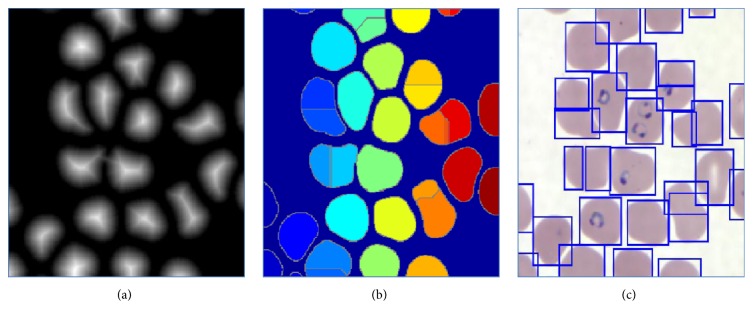
Output obtained after watershed transform (a), distance transform (b), and watershed output (c) after applying major and minor axis criteria.

The segmentation is carried out as follows:

(i) Distance transform is calculated using Euclidean distance formula:
  (1) $$\begin{array}{c} Distance=\sqrt{{\left(x_{1}-x_{2}\right)}^{2}{+\left(y_{1}-y_{2}\right)}^{2}}, \end{array}$$
 where “*a*” and “*b*” are the two neighboring pixels between which the distance is calculated and their position can be given as *a*(*x*_1_, *y*_1_) and *b*(*x*_2_, *y*_2_), where *x*_1_, *x*_2_ are the *x*-coordinates and *y*_1_, *y*_2_ are *y*-coordinates of the two neighboring pixels which are considered. Then, these distance values are calculated for all the pixels in an image and a distance matrix is formed. This is used as an input for the watershed transform.
(ii) After calculating the distance between each pixel, it creates matrix of these values called label matrix.
(iii) Label matrix is then given as an input to watershed and the image is obtained as shown in Figures 7(a) and 7(b).
(iv) This obtained image is segmented using the major and minor axis criteria as shown in Figure 7(c).

### 2.3. Feature Extraction

#### 2.3.1. Statistical Features

The staining of the slides varies in the color acquired by the RBC, in different colors like blue, purplish, pink, and so forth [4]. Hence the color intensity varies, therefore that can be counted into the feature list, and this variation can be plot on histogram of each image. The histogram is studied using the statistical values of color variation of the image on the basis of different staining. Therefore, the histogram based statistical features are studied in this research. The variance feature gives the variation of the intensity values in image, while the skewness gives the symmetric distribution of those intensity values. There are high peaks observed in the infected cells as compared to the normal cells, and hence the kurtosis values are efficient for detection of infected red blood cells. The statistical features are calculated as described below.

If image function is *I*(*x*, *y*) of two space variables *x* and *y*, *x* = 0,1, 2,…, *N* − 1 and *y* = 0,1, 2,…, *M* − 1, then the discrete values of function *I*(*x*, *y*) can be taken in terms of the intensity values defined by “*k*” as *k* = 0,1, 2,…, *G* − 1, where *G* represents the total number of intensity levels in the image. All these features are calculated using the histogram of the image *h*(*k*) and probability density function *p*(*k*). Here *p*(*k*) is the ratio of all the intensity values *h*(*k*) and the total number of pixels in the image. The features are calculated as follows:

(2) Mean:  μ=∑k=0G−1kpk,Variance:  σ2=∑k=0G−1k−12pk,Skewness:  μ3=σ−3∑k=0G−1k−μ3pk,Kurtosis:  μ4=σ−4∑k=0G−1k−μ4pk−3.

See [9].

#### 2.3.2. Texture Based Features

In second stage, that is, schizocytes, there is growth of granular spotting, that is, the growth of cytoplasm known as matured trophozoites. These cytoplasm dots create a particular texture which differentiates it from other two stages more prominently. Hence, the texture features are included in the feature extraction list. The infected cells are correlated with the background pixels which can be defined by the correlation values calculated and the energy level of those infected cells defines them with higher energy values as compared to normal cell values.

The texture based features are calculated using Gray-Level Cooccurrence Matrix (GLCM) from the input. The GLCM calculates the occurrence of a pixel with gray-level value “*i*” horizontally adjacent to a pixel with the value “*j*.” Every element can be mentioned by (*i*, *j*) in GLCM which specifies the number of times that the pixel with a value “*i*” occurred horizontally adjacent to a pixel with value “*j*.”

These texture based features can be calculated and used as follows:

(i) * Contrast* is the value which returns the measure of intensity contrast between a pixel and the neighboring pixel over the complete image. The standard range of these values for general images is [0, (size(GLCM, 1) − 1^2^)] [10]:
  (3) $$\begin{array}{c} Contrast=\underset{i,j}{\sum }{\left|\;i-j\;\right|}^{2}p\left(\;i,j\;\right). \end{array}$$
(ii) * Correlation* is the measure of how pixel is correlated to the neighboring pixel in the complete image. The standard range of these values for general images is [−1, 1] [10]:
  (4) $$\begin{array}{c} Correlation=\underset{i,j}{\sum }\frac{\left(i-\mu i\right)\left(j-\mu j\right)p\left(i,j\right)}{\sigma_{i}\sigma_{j}}. \end{array}$$
(iii) * Energy* is the sum of all squared elements in the GLCM matrix. The standard range of these values for general images is [0, 1] [10]:
  (5) $$\begin{array}{c} Energy=\underset{i,j}{\sum }{p\left(i,j\right)}^{2}, \end{array}$$
 where the Gray-Level Cooccurrence Matrix (GLCM) is calculated first and then the properties, like contrast, correlation, and energy, are calculated.

**Figure 8:**
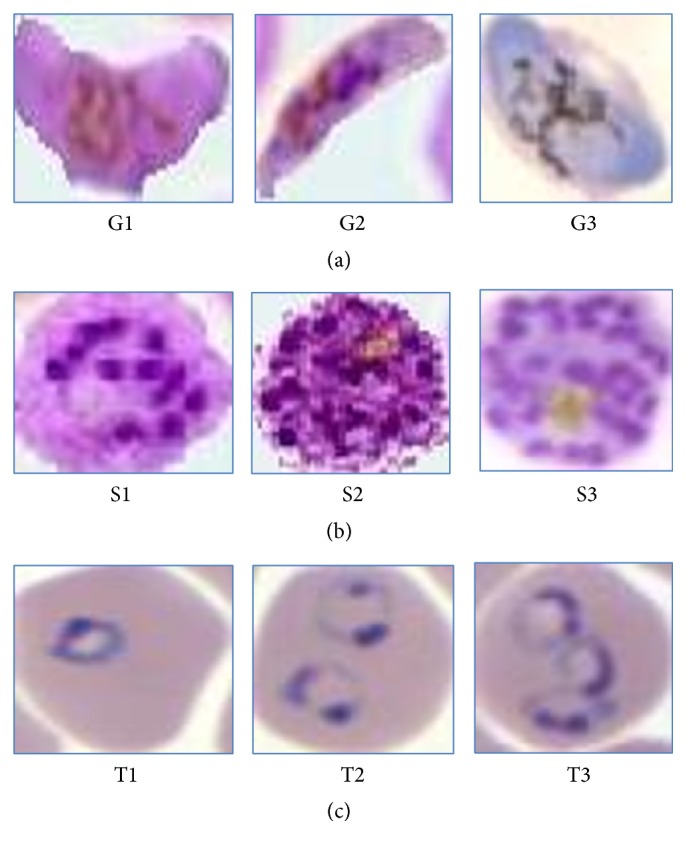
Samples of training images.

The different samples of all three stages of malaria parasite are taken for the training of the Support Vector Machine classifier as shown in Figure 8. The three images in (a) are of third stage as gametocytes while (b) consists of second stage, that is, the schizonts, and (c) consists of the first stage trophozoites image samples which are used for training purpose.

The different values for all seven features are given to the SVM for training the stages. The images used are labeled according to the stages name as the trophozoites are named as T1, T2, T3,…, and so forth, while schizonts are named as S1, S2, S3,…, and so forth, and lastly third-stage gametocytes are named as G1, G2, G3,…, and so forth. The values of features are as shown in Tables 1, 2, and 3 for each stage I, II, III, respectively.

### 2.4. Image Classification and Parasitemia Estimation

Classification is done using the feature matrix created by calculating all the feature values for each RBC. This feature matrix of trained data is compared with the feature matrix for the image taken as an input. Then, the class is decided accordingly on the comparison results and displayed. The Support Vector Machine (SVM) is for the classification. SVM is a supervised classifier which requires trained dataset and can be used for multistage classification using multi-SVM.

Based on the classification results obtained in the work, the quality of the result is to be decided by calculating as follows:

(6) Accuracy=TP+TNTP+TN+FP+FN,Sensitivity=TPTP+FN,Specificity=TNFP+TN

(see [11]), where TP is true positive (infected sample correctly classified), TN is true negative (normal sample correctly classified), FP is false positive (normal sample misclassified), and FN is false negative (infected sample misclassified).

All the values of calculation done for the above values are as shown in Table 4 with the elapse time requirement. The algorithm is executed on hardware platform of Laptop, model: Lenovo Win7PC, Core i5 @250 GHz, RAM: 8 GB, 64-bit OS. From this, it is observed that the time required for calculation is proportional to the number of RBC in the image. All the images used for testing were labeled as Ts-1, Ts-2, Ts-3,…, and so forth, where Ts stands for testing sample and the number of sample followed by this.

**Figure 9:**
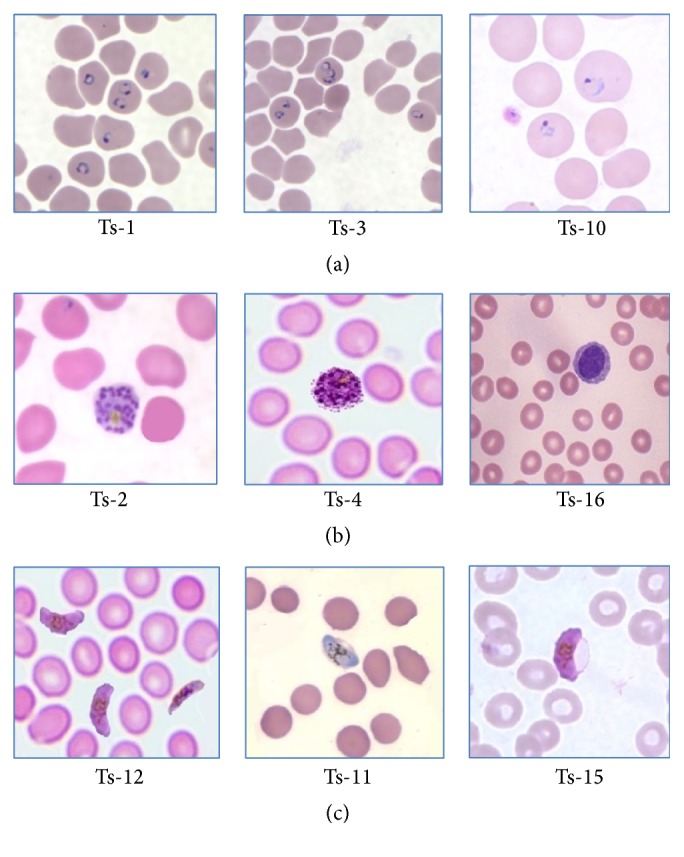
Sample images from test database used.

Some of the testing samples are shown in Figure 9 where in (a) there are first-stage sample images, that is, trophozoites, and (b) consists of second-stage sample images, that is, schizonts, and lastly (c) consists of all the third-stage parasite samples, that is, gametocytes.

## 3. Experimental Results

The complete process is performed as mentioned stepwise above by considering all the statistical features and texture based features and then obtained the results in terms of accuracy; sensitivity and specificity are calculated in percentage. Secondly the statistical features were kept constant and texture features were considered one by one individually and calculated the values for accuracy, sensitivity, and specificity for the same database as mentioned before.

**Figure 10:**
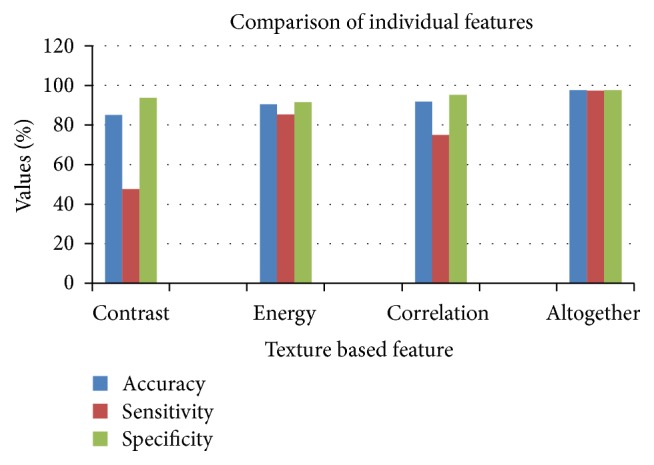
Comparison of individual texture based features in terms of accuracy, sensitivity, and specificity.

Table 5 gives all the results obtained after calculating the values of accuracy, sensitivity, and specificity for individual texture based features and altogether with the statistical features this comparison is shown in a graphical way in Figure 10.

## 4. Results and Discussion

From the observation done in Table 5 for accuracy, sensitivity, and specificity for considering all features together and for individual texture based features, it can be seen that if all the features are used together, then the high diagnosis accuracy can be achieved. If all the features are used together, the SVM gets trained very efficiently and the classification of the parasitic and nonparasitic samples and the stages determination are done with higher accuracy of about 97%. This means the diagnosing of parasite and the detection of stage are more reliable if all the features are used together. Among three texture based features, namely, contrast, energy, and correlation, when only energy features are considered for classification, it gives more accuracy, sensitivity, and specificity up to 90.55%, 85.36%, and 91.66%, respectively. The high accuracy achieved by presented system can be compared with other methods in the literature as shown in Table 6.

## 5. Conclusion

As the number of people infected by malaria in tropical region is high, there is special need of early diagnosis of malaria parasite, for its prevention against getting into next stage and medication of a person. This paper has presented automated diagnosis of malaria parasite in blood images. The diagnosis is done by using features like statistical and textural based features which has enhanced the diagnosis accuracy of the presented system. Additionally the presented method is also helpful in detecting stages of malaria parasite. The segmentation is done using watershed algorithm using distance transform which counts the overlapped RBC in the blood images. The system achieved high accuracy of 97.7% for automatic diagnosis of malaria parasite in blood images.

Further this technique can be used for diagnosing different hematological disorders by training the SVM accordingly. The textural properties can be used for enhancing the results of accuracy and sensitivity.

## Figures and Tables

**Table 1:** The values obtained while training dataset for stage I.

Stage I: trophozoites							
Image number	Statistical features				Texture based features		
	Mean	Variance	Skewness	Kurtosis	Contrast	Energy	Correlation
T1	0.950	0.006	−2.052	−25.114	0.059	0.843	0.859
T2	0.951	0.007	−3.339	−38.326	0.053	0.868	0.906
T3	0.961	0.006	−5.414	−68.288	0.054	0.938	0.894
T4	0.937	0.013	−2.647	−22.71	0.092	0.792	0.918
T5	0.946	0.013	−3.281	−28.756	0.097	0.846	0.913

**Table 2:** The values obtained while training dataset for stage II.

Stage II: schizonts							
Image number	Statistical features				Texture based features		
	Mean	Variance	Skewness	Kurtosis	Contrast	Energy	Correlation
S1	0.961	0.004	−1.055	−15.137	0.0269	0.766	0.885
S2	0.772	0.107	−0.308	−0.940	0.397	0.203	0.937
S3	0.812	0.085	−0.449	−1.539	0.377	0.272	0.929
S4	0.785	0.100	−0.359	−1.133	0.381	0.228	0.938
S5	0.621	0.230	0.015	0.0317	1.144	0.106	0.885

**Table 3:** The values obtained while training dataset for stage III.

Stage III: gametocytes							
Image number	Statistical features				Texture based features		
	Mean	Variance	Skewness	Kurtosis	Contrast	Energy	Correlation
G1	0.835	0.055	−0.194	−0.825	0.242	0.267	0.915
G2	0.974	0.001	−1.758	−41.813	0.008	0.926	0.887
G3	0.830	0.056	−0.157	−0.661	0.188	0.256	0.933
G4	0.897	0.043	−1.347	−6.491	0.201	0.544	0.942
G5	0.945	0.013	−2.679	−22.707	0.203	0.753	0.823

**Table 4:** Calculations of total RBC, infected RBC, and the TP, TN, FP, and FN values considering all the seven features.

Image number	Total RBC count		Infected RBC count						
	Manual	Algorithm	Manual	Algorithm	TP	TN	FP	FN	Elapsed time
Ts-1	28	29	4	5	4	24	1	0	33.94
Ts-2	11	10	1	1	1	10	0	0	21.96
Ts-3	30	33	3	3	3	27	0	0	34.19
Ts-4	14	15	1	1	1	14	0	0	23.97
Ts-5	10	10	4	4	4	6	0	0	21.57
Ts-6	10	10	3	3	3	7	0	0	20.82
Ts-7	11	11	3	4	3	8	1	0	21.00
Ts-8	7	7	1	1	1	6	0	0	17.15
Ts-9	12	11	3	3	3	8	0	0	20.72
Ts-10	12	12	1	2	1	11	1	0	21.36
Ts-11	22	25	3	4	3	23	1	0	30.84
Ts-12	22	25	3	5	2	23	1	1	30.69
Ts-13	8	10	5	5	5	5	0	0	19.69
Ts-14	15	16	3	3	3	13	0	0	23.95
Ts-15	36	37	1	1	1	36	0	0	31.83
Total	248	261	39	45	38	221	5	1	24.91

**Table 5:** Comparison of individual texture based features in terms of accuracy, sensitivity, and specificity.

	Contrast%	Energy%	Correlation%	Altogether%
Accuracy	85.1	90.55	91.8	97.7
Sensitivity	47.7	85.36	75	97.4
Specificity	93.7	91.66	95.3	97.7

**Table 6:** Summary of comparison with other works.

Sr. number	Author	Reference number	Features used	Accuracy
1	Ross et al.	[12]	Morphological (erosion, dilation)	85.3%
2	Tek et al.	[13]	Color and cell geometry (Hough transform for circle detection)	83%
3	Das et al.	[8]	Morphological (opening, closing)	88.77%
4	Kareem	[14, 15]	Edge filtering (Sobel, Canny)	86%
5	Savkare and Narote	[9]	Statistical features (area, mean, and variance)	94%
6	Alomari et al.	[16]	Geometry (modified randomized circle detection)	95%
7	Presented system	[17]	Texture based and statistical based	97.7%
